# An Outpatient Cross-Sectional Study of Physicians’ Empathy From the Patients’ Perspective: A Comparison of the Arabic Version of the Consultation and Relational Empathy (CARE) Measure Across Four Disciplines

**DOI:** 10.7759/cureus.101048

**Published:** 2026-01-07

**Authors:** Eiad A AlFaris, Abdullah M Ahmed, Gominda Ponnamperuma, Lemmese Alwatban, Hamad A Alkhenizan, Bandar H Alaamer, Faisal M AlRashed, Bassam A Alghizzi, Ahmed S AlGhamdi, Abdullah S AlGhamdi

**Affiliations:** 1 Medical Education Research and Development, King Saud University, Riyadh, SAU; 2 Department of Family and Community Medicine, College of Medicine, King Saud University, Riyadh, SAU; 3 Department of Medical Education, Faculty of Medicine, University of Colombo, Colombo, LKA; 4 University Family Medicine Center, King Saud University, Riyadh, SAU; 5 College of Medicine, King Saud University, Riyadh, SAU

**Keywords:** care instrument, empathy differences, family medicine, perceived empathy by patients, physician empathy

## Abstract

Purpose

Empathy plays a crucial role in the patient-physician relationship and is often regarded as a key determinant of effective medical care. This study aimed to validate the Arabic version of the Consultation and Relational Empathy (CARE) measure and compare patients' perceptions of physician empathy across four medical specialties.

Materials and methods

A cross-sectional, survey-based design was employed. Participants were randomly selected from family medicine, general surgery, internal medicine, and orthopedics clinics (36 patients per specialty). The investigators individually assisted each patient in completing the Arabic version of the CARE measure, which measured the level of empathy perceived during their medical consultation. After translation, a validation study for the Arabic version was conducted. The mean CARE scores across the four specialties were analyzed using nonparametric tests, namely the Mann-Whitney U test and the Kruskal-Wallis test, with statistical significance set at p < 0.05.

Results

A significant association was found between physicians' specialties and patients' perceptions of empathy levels (p = 0.047). Family medicine physicians had the highest mean empathy scores, while orthopedic surgeons had the lowest. Patients’ marital status was significantly associated with their perception of their doctors’ empathy (p = 0.032), with married patients reporting the highest level of empathy and single patients the lowest. The (CARE) measure demonstrated high reliability, with a Cronbach’s alpha of 0.926.

Conclusions

Patients' perceptions of doctors' empathy were significantly higher among family medicine physicians than among orthopedic surgeons. It shows that the Arabic version of the CARE measure has strong psychometric properties and is valid for use in similar outpatient settings. The results indicate the need to enhance the training curricula of medical programs in general and surgical programs in particular.

## Introduction

Empathy is the capacity to enter into another person’s experience. For the physician, it is the capacity to sense what it is like to be the patient: to experience illness, disability, depression, and so on [1]. It involves understanding, feeling, sharing, and self-other differentiation [2]. Unlike sympathy, which is an emotional reaction, empathy requires cognitive understanding and perspective-taking [3,4]. It enables healthcare professionals to perceive illness from the patient’s point of view and communicate understanding effectively [1,5]. Despite its importance, around 70% of health professionals report challenges in developing empathy, often influenced by factors such as age, experience, stress, and burnout [6].

In terms of benefits to patients, empathy is associated with successful remedial actions, reduced patient pain, improved prognostic accuracy, increased patient satisfaction and compliance with advice and treatment, improved clinical outcomes, greater insight into safety, and reduced anxiety and depression [7-9].

Empathy also improves treatment adherence, reduces patient stress, and leads to better health outcomes in chronic conditions like diabetes and cancer [3]. It is known that patients may get discouraged from seeking medical therapy when minimal empathy is shown [10,11].

It also benefits physicians by increasing job satisfaction and diagnostic accuracy and reducing burnout and malpractice risk. Studies further link empathy to prosocial and altruistic behaviors [3]. It can also reduce the risk of medical malpractice, enhance connections and trust among colleagues, and protect against exclusion from work [7-9].

However, barriers such as time constraints, heavy workloads, and limited empathy training persist in restraining empathetic behavior [6]. It was found in several studies that patients may get discouraged from seeking medical therapy when minimal empathy was shown [10,11]. With this knowledge, a greater emphasis on empathy in the training of doctors is essential.

The Consultation and Relational Empathy (CARE) measure [12] is a patient-rated tool that assesses healthcare providers' interpersonal skills and empathy levels [11]. Most surgeons in a Canadian study found the CARE measure relevant to their practice and a useful tool, and some reported an intention to change their practice based on the results. The positive reception from surgeons suggests that it is a valuable tool for providing feedback and encouraging better communication [13].

Empathy has gained increasing attention in Saudi healthcare research. Alzayer et al. examined factors associated with patient-perceived physician empathy in a primary care setting. While these studies have provided important foundational insights, further work is needed to evaluate the instrument across diverse clinical specialties and to explore the demographic and professional factors associated with patient-perceived empathy in broader Saudi healthcare contexts [10]. To address this gap, the present study has two aims: the first is to assess the psychometric properties of the CARE measure in a multi-specialty outpatient environment. The second is to investigate variations in empathy perceptions across physician specialties and patient sociodemographic characteristics. By combining validation and application of the CARE measure in a real-world clinical setting, this study extends prior Saudi research. It contributes novel evidence supporting the use of empathy assessment tools in routine practice, thereby fostering improvements in patient-centered care delivery in Saudi Arabia.

## Materials and methods

Study design

This research employed a cross-sectional survey-based approach. The study was conducted from September 1 to December 30, 2022. The target population included Arabic-speaking, male and female patients who visited the outpatient clinics at King Khalid University Hospital in Riyadh, Saudi Arabia.

Sampling

A stratified random sampling method was used to select patients from four medical specialties, namely family medicine, general surgery, internal medicine, and orthopedics.

Sample size calculation

For factor analysis, the widely accepted minimum sample size recommendation of 10 subjects per variable (questionnaire item) was used [14]. To determine the minimum number of subjects required for adequate study power, we used ClinCalc (ClinCalc LLC, Arlington Heights, IL, USA) [15]. Going by the mean score of 33 for the first group in Hong Kong [16] and 43 for the second group in the United Kingdom [16], and a standard deviation of 9.0 with an alpha of 0.05 and a power of 80%, the estimated sample size was 26 per group. Allowing for 10% possible participant noncompliance, we sampled 36 patients from each of the four clinics above, yielding a total of 144 participants. Only individuals aged 18 years or older and Arabic speakers were eligible for inclusion. Patients younger than 18 and non-Arabic-speaking patients were excluded.

Measurement tool

The CARE measure evaluates physician performance based on ten key questions, addressing aspects such as “active listening” and “demonstrating care and compassion”. Responses were recorded using a five-point scale, ranging from “poor” (1 point) to “excellent” (5 points). The total empathy score, calculated by summing responses across all ten questions, ranged from 10 to 50, with higher scores reflecting greater empathy [12].

Translation and validation

We could not use the Arabic version of an earlier study by Al-Habbal et al. [17] because, at the time of conducting and planning this research, that study had not yet been published. To ensure clarity, the original English version of the CARE measure was translated into Arabic by two bilingual physicians. To verify the accuracy of the translation, a back-translation into English was performed by another physician who had no prior exposure to the instrument. The translated and back-translated versions were highly consistent. The translated version was piloted with a group of patients, similar to the study participants. They were able to read and understand the phrases of the measure without assistance. This translated Arabic version of the CARE measure (Appendices) was utilized for data collection.

Ethical considerations and consent

All participants were able to read the consent form. They were initially asked whether they were willing to participate and were informed that refusal would not affect the medical care they received at the clinics. Participants were assured of confidentiality and that all collected data would be used solely for research purposes. Upon confirming their willingness to participate, written informed consent was obtained from each participant. Ethical approval for the study was granted by the Institutional Review Board of the College of Medicine of King Saud University (approval number: 22/0596/IRB) on August 10, 2022.

Data collection

The CARE measure was administered to patients by trained third-year medical students as part of their community medicine course. In addition to the CARE measure, demographic data were recorded, including the patient's sex, age, educational background, nationality, marital status, place of residence, occupation, and income.

Data analysis

In the psychometric validation study, the Kaiser-Meyer-Olkin (KMO) test, a measure of sampling adequacy, and Bartlett's test of sphericity, a measure of the factorability of the results, were used to assess the assumptions of factor analysis. Exploratory factor analysis was performed to determine whether the translated version yielded a factor structure (i.e., a one-factor solution) similar to that of the original CARE measure [13]. Following validation, categorical variables were analyzed as percentages, while continuous data were analyzed as means, standard deviations, and scores. Normality was examined for continuous measures prior to proceeding with data analysis, and it was found to be negatively skewed (not normally distributed). Therefore, it was decided to use non-parametric tests to compare mean empathy scores. The Mann-Whitney U test was used for two-group comparisons (e.g., patients’ sex, educational level). In contrast, the Kruskal-Wallis test was used for comparisons involving more than two groups (e.g., the doctors’ specialties, marital status, and age groups). Statistical significance was set at p < 0.05.

## Results

The total study sample consisted of 144 participants, with a 100% response rate, and males comprised 75 (52.1%) of the group. The average age of the participants was 44.8 ± 15.7 years, ranging from 18 to 80 years. The majority of patients were Saudi nationals (137, 95.1%) and married (94, 65.3%). More than half (77, 53.5%) had attained a university degree or higher. Regarding employment status, nearly half of the participants were employed (68, 47.2%), while the rest were either unemployed (32, 22.2%), retired (18, 12.5%), or housewives (26, 18.1%) (Table 1).

**Table 1:** Distribution of the study participants according to different sociodemographic categories SD: standard deviation

Characteristic		N	%
Age mean (SD) = 44.76 (15.6)	18-39	58	40.3%
	40-59	55	38.2%
	60-80	31	21.5%
Sex	Male	75	52.1%
	Female	69	47.9%
Education	High school and lower	67	46.5%
	University and higher	77	53.5%
Marital status	Married	94	66.2%
	Single	36	24.6%
	Divorced/widowed	14	9.1%
Job	Employed	68	47.2%
	Unemployed	32	22.2%
	Retired	18	12.5%
	Housewife	26	18.1%
Specialty	Family medicine	36	25%
	Orthopedic	36	25%
	Internal medicine	36	25%
	Surgery	36	25%

Regarding the rating of the score distribution, none of the items had missing values, and only items 9 (3.5%) and 10 (4.9%) had responses in the "does not apply" category. For all 10 items, the most frequent rating was excellent, ranging from 90% to 78% (Table 2).

**Table 2:** Distribution of the responses to the 10 items of the Arabic CARE measure on the five-point scale ranging from "poor" (1 point) to "excellent" (5 points) CARE: Consultation and Relational Empathy

Item	Poor (%)	Fair (%)	Good (%)	Very good (%)	Excellent (%)	Does not apply (%)	Missing (%)
1	0 (0)	3 (2.1)	6 (4.2)	4 (2.8)	130 (90.3)	1(0.7)	0 (0)
2	0 (0)	6 (4.2)	5 (3.5)	11 (7.9)	122 (84.7)	0 (0)	0 (0)
3	1 (0.7)	1 (0.7)	7 (4.9)	8 (5.6)	127 (88.2)	0 (0)	0 (0)
4	1 (0.7)	2 (1.4)	5 (3.5)	12 (8.3)	123 (85.4)	1 (0.7)	0 (0)
5	1 (0.7)	3 (2.1)	7 (4.9)	17 (11.8)	115 (79.9)	1 (0.7)	0 (0)
6	3 (2.1)	1 (0.7)	10 (6.9)	9 (6.3)	121 (84)	0 (0)	0 (0)
7	2 (1.4)	1 (0.7)	7 (4.9)	13 (9)	121 (84)	0 (0)	0 (0)
8	1 (0.7)	3 (2.1)	4 (2.8)	12 (8.3)	123 (85.4)	1 (0.7)	0 (0)
9	1 (0.7)	3 (2.1)	3 (2.1)	19 (13.2)	113 (78.5)	5 (3.5)	0 (0)
10	0 (0)	2 (1.4)	7 (4.9)	15 (10.4)	113 (78.5)	7 (4.9)	0 (0)

The CARE measure demonstrated high reliability, with a Cronbach’s alpha of 0.926. The KMO measure of sampling adequacy was 0.878, and Bartlett's test of sphericity indicated a factorizable correlation matrix (chi-square (45) = 1222.3; p = 0.000). After analysis of the sedimentation plot and eigenvalues (all >1), a 1-factor solution explaining 65.3% of the total variance was identified. This factor structure was the same as the one-factor solution (i.e., the original CARE measure) that the original CARE measure produced (Table 3).

**Table 3:** Factor analysis of the 10 items of the Arabic CARE questionnaire: Cronbach's alpha and factor loadings CARE: Consultation and Relational Empathy, CI: confidence interval

Item	Scale mean if item deleted	Corrected item-total correlation	Cronbach's alpha if item deleted	95% CI for Cronbach’s alpha if item deleted	Factor loadings
Making you feel at ease	42.17	0.687	0.921	0.900-0.939	0.772
Letting you tell your story	42.24	0.835	0.914	0.891-0.933	0.891
Really listening	42.17	0.867	0.914	0.892-0.934	0.913
Being interested in you as a whole person	42.22	0.828	0.914	0.891-0.933	0.873
Fully understand your concerns	42.31	0.787	0.915	0.893-0.934	0.836
Showing care and compassion	42.27	0.756	0.917	0.895-0.936	0.818
Being positive	42.23	0.791	0.916	0.894-0.935	0.858
Explaining things clearly	42.23	0.662	0.922	0.901-0.939	0.709
Helping you take control	42.40	0.723	0.921	0.900-0.939	0.775
Making a plan of action with you	42.45	0.531	0.937	0.920-0.951	0.582

The overall mean CARE score across all specialties was 46.97, with family medicine physicians scoring the highest at 48.33 and orthopedic surgeons scoring the lowest at 44.47. Patients' perceptions of empathy differed significantly with physicians' specialty (Kruskal-Wallis test, p = 0.002, Z = 15.1). Patients’ sex, age, and education did not show a statistically significant association with their perceptions of physicians’ empathy. Patients’ marital status was significantly associated with their perception of their doctors’ empathy (p = 0.032, Z = 6.86), with married patients reporting the highest level of empathy and single patients the lowest. Patients' perceived empathy scores did not change significantly when seen by male or female physicians (Table 4).

**Table 4:** Association between patients’ perceived physician empathy score and patients’/doctors’ sociodemographic variables * Mann-Whitney test, ** Kruskal-Wallis test

Variables		n	Mean rank	^*^U/ ^**^Z -value^*^	p-value
Patients’ sex	Male	75	72.3	-0.087*	0.931
	Female	69	72.8		
Doctors’ sex	Male	75	71.2	-1.046	0.296
	Female	69	79.9		
Patients’ education	High school and lower	67	76.3	-1.16*	0.26
	University and higher	77	69.2		
Doctors’ specialty	Family medicine	36	82.8	15.1**	0.002
	Orthopedic	36	52.8		
	Internal medicine	36	80.5		
	Surgery	36	73.8		
Patients’ age	18-39	58	65.4	4.3**	0.115
	40-59	55	74.8		
	60-80	31	81.5		
Patients’ marital status	Married	94	77.6	6.86**	0.032
	Single	36	58.9		
	Divorced/widowed	14	72.6		
Patients’ job	Employed	68	68.3	4.1**	0.255
	Unemployed	32	68.6		
	Retired	18	82.6		
	Housewife	26	81.1		

## Discussion

This study's translation of the CARE measure demonstrated strong psychometric properties, similar to those of Al-Habbal et al.'s version [17], supporting its reliability and construct validity for use in clinical practice in Arabic countries. However, we recommend that future research use the Arabic version of Al-Habbal et al.'s study [17] rather than the current study's translation, as recommended by the copyright holder, Professor Stewart Mercer. Additionally, the findings revealed significant variation in empathy scores across four medical specialties; family physicians had the highest mean empathy score, and orthopedic surgeons had the lowest. The finding of higher empathy scores toward their doctors among married patients than among single patients differs from that in the study of Korean patients with chronic diseases [18].

The overall mean CARE score of 46.9 observed in this study is higher than those reported in numerous other studies. A systematic review encompassing 15 countries reported a mean score of 40.48 [16]. Similarly, the mean empathy scores across various settings were 42.6 in a pediatric orthopedics clinic in the USA [11] and 44.5 in a vaccination counseling clinic in Italy [19]. Higher empathy scores were recorded in Australia (44.88 [16]) and in Austin, the United States (46 [20]). It is worth noting that the latter study involved patients seen by five orthopedic surgeons in their outpatient offices [20], although in the present study, orthopedic surgeons' scores were the lowest. Across settings, mean empathy scores varied, e.g., in the United Kingdom (43.07) [16] and Lebanon (36.12) [17], while the lowest scores were reported in Hong Kong (33.46) [16] and Ethiopia (31.34) [21].

The observation that family medicine physicians exhibited significantly higher empathy scores than orthopedic surgeons aligns with the findings from multiple studies [9,10,13,22]. A systematic review investigating empathy in compassionate care suggested that physicians in patient-centered specialties demonstrated higher empathy towards their patients than those in technology-driven or surgical fields [23]. However, an alternate systematic review found no significant difference in CARE scores between primary care physicians and specialists [16]. Regarding doctors’ gender, the current study did not reveal a significant disparity in patients’ empathy scores when seen by male or female physicians. While several studies have reported higher patient-perceived empathy among female physicians than among their male counterparts [13,16,23], others have found no gender-related differences among physicians, and a few studies even indicate higher empathy scores among male physicians [23]. It is possible that socio-cultural factors and patient expectations regarding physicians’ gender influenced these findings.

Strengths and limitations

The current study sample was collected using stratified random sampling, and the results showed a high reliability score (Cronbach's alpha = 0.926), which may have improved internal comparisons across specialties but does not necessarily confer broad external generalizability. The Arabic version of the CARE measure was validated and showed favorable psychometric properties. The use of medical students, rather than independent research staff, to administer the survey may have introduced social desirability and courtesy bias, particularly in cultural contexts characterized by strong respect for clinicians. This may have led to artificially elevated empathy scores and compromised the validity of the findings. Although efforts were made to mitigate this effect by ensuring participant anonymity and providing neutral instructions, this limitation should be considered when interpreting the results. Furthermore, the sample was confined to the outpatient clinics of a single hospital, limiting generalizability.

## Conclusions

The study highlights how strongly patients value empathy in their clinical encounters and shows that the Arabic version of the CARE measure has strong psychometric properties and is valid for use in similar outpatient settings. Across the four medical specialties examined, patients consistently perceived family physicians as showing the highest levels of empathic care, while orthopedic surgeons received the lowest ratings. The finding that married patients tended to rate physicians as more empathetic than single patients hints at the influence of personal and social factors on the patient-physician relationship. By emphasizing empathic practice as a core clinical competency, healthcare institutions can help foster more positive patient experiences and interpersonal skills.

## Appendices

النسخة المترجمة من مقياس الاستشارة والتعاطف

1. الجنس: □ ذكر □ أنثى

2. العمر (بالسنين): ……………………

3. اسم الطبيب: ………………………………………………………………

4. الحالة التعليمية: □ أمي □ لم يكمل الابتدائي □ الابتدائي □ المتوسط

□ الثانوية العامة □ دبلوم □ البكالوريوس □ الماجستير

□ الدكتوراه

5. الجنسية / العرق: □ سعودي □ غير سعودي

6. الحالة الاجتماعية: □ متزوج □ أعزب □ مطلق □ أرمل □ لا أرغب بالإجابة

7. مكان الإقامة: □ الرياض □ خارج الرياض □ خارج المملكة

8. الوظيفة: ………………………………………………

9. مقياس الاستشارة والتعاطف

**Table 5:** مقیاس الاستشارة والتعاطف This table presents the questionnaire administered to subjects at a tertiary care center. The questionnaire consists of 10 items.

رجاءًا قيم العبارات التالية عن زيارتك اليوم. ضع علامة √ في المربع المناسب لكل عبارة من العبارات التاليه							
لا ينطبق	ممتاز	جيد جدًا	جيد	مقبول	سيء	ما رأيك بالطبيب من حيث التالي...	
						جعلك تشعر بالارتياح... (تعامل معك بمودة ولطف واحترام، ولم يكن فظً التعامل)	1
						اعطائك المجال للحديث بحرية... (هل ترك المجال لك لشرح حالتك، دون مقاطعة)	2
						الإنصات إليك... (الاستماع لك باهتمام ، وعدم الانشغال بأمر آخر)	3
						إبداء اللإهتمام الكافي... (يسأل عن تفاصيل لها صلة بحالتك, ويعاملك كإنسان؛ وليس كمجرد حالة في ملف)	4
						إدراكه التام بمخاوفك... (يُظهر لك بأنه يفهم معاناتك, ومسببات قلقك) (هل لديه ادراك لمخاوفك واسباب قلقك؟)	5
						تعاطفه معك ورعايته لك... (يبدو قلقا على صحتك، ويتواصل معك باحترام)	6
						الإيجابية في التعامل والاحترافية في التواصل... (التزامه الايجابية معك بغض النظر عن حالتك, والتواصل معك بكل احترافية ووضوح, وبدون تردد)	7
						الشفافية والوضوح... (يعطيك الإجابات الوافية عن استفساراتك, والشرح الكافي لكل تفاصيل حالتك, وعدم إخفاء أي أمر له أهمية)	8
						مساعدتك في التعامل مع مرضك... (تأثيره الايجابي وتشجيعه في ما يخص مرضك, وإعطائك السبل الكفيلة بتحسين وضعك الصحي والنفسي ,دون أمرك بفعل معين)	9
						تحديد الخطه العلاجيه الأنسب لك... (مناقشته لك بالخيارات المتوفرة بالقدر الكافي, والتفاهم سويًا حول القرارات ذات التأثير الأكبر على)	10

**Table 6:** CARE measure English translation CARE: Consultation and Relational Empathy

Please rate the following statements about today’s consultation. Please tick one box for each statement and answer every statement.							
How was the doctor a…		Poor	Fair	Good	Very good	Excellent	Does not apply
1	Making you feel at ease… (being friendly and warm towards you, treating you with respect; not cold or abrupt)						
2	Letting you tell your “story"… (giving you time to fully describe your illness in your own words; not interrupting or diverting you)						
3	Really listening… (paying close attention to what you were saying; not looking at the notes or computer as you were talking)						
4	Being interested in you as a whole person… (asking/knowing relevant details about your life, your situation; not treating you as “just a number”)						
5	Fully understanding your concerns… (communicating that he/she had accurately understood your concerns; not overlooking or dismissing anything)						
6	Showing care and compassion… (seeming genuinely concerned, connecting with you on a human level; not being indifferent or “detached”)						
7	Being positive… (having a positive approach and a positive attitude; being honest but not negative about your problems)						
8	Explaining things clearly… (fully answering your questions, explaining clearly, giving you adequate information; not being vague)						
9	Helping you to take control… (exploring with you what you can do to improve your health yourself; encouraging rather than “lecturing” you)						
10	Making a plan of action with you… (discussing the options, involving you in decisions as much as you want to be involved; not ignoring your views)						

## References

[1] Freeman Freeman, Thomas R ( 2016). McWhinney's Textbook of Family Medicine (4 edn).

[2] Håkansson Eklund J, Summer Meranius M (2021). Toward a consensus on the nature of empathy: a review of reviews. Patient Educ Couns.

[3] Moudatsou M, Stavropoulou A, Philalithis A, Koukouli S (2020). The role of empathy in health and social care professionals. Healthcare (Basel).

[4] Walsh S, O'Neill A, Hannigan A, Harmon D (2019). Patient-rated physician empathy and patient satisfaction during pain clinic consultations. Ir J Med Sci.

[5] Rathert C, Wyrwich MD, Boren SA (2013). Patient-centered care and outcomes: a systematic review of the literature. Med Care Res Rev.

[6] Klimecki OM (2019). The role of empathy and compassion in conflict resolution. Emotion Review.

[7] Katsari V, Tyritidou A, Domeyer PR (2020). Physicians’ self-assessed empathy and patients’ perceptions of physicians’ empathy: validation of the Greek Jefferson Scale of patient perception of physician empathy. Biomed Res Int.

[8] Bunin J, Shohfi E, Meyer H, Ely EW, Varpio L (2021). The burden they bear: a scoping review of physician empathy in the intensive care unit. J Crit Care.

[9] Derksen F, Bensing J, Lagro-Janssen A (2013). Effectiveness of empathy in general practice: a systematic review. Br J Gen Pract.

[10] Alzayer ZM, Abdulkader RS, Jeyashree K, Alselihem A (2019). Patient-rated physicians' empathy and its determinants in Riyadh, Saudi Arabia. J Family Community Med.

[11] Singleton IM, Garfinkel RJ, Malone JB, Temkit MH, Belthur MV (2022). Perceived physician empathy in pediatric orthopedics: a cross-sectional study. J Patient Exp.

[12] Mercer SW, Maxwell M, Heaney D, Watt GC (2004). The consultation and relational empathy (CARE) measure: development and preliminary validation and reliability of an empathy-based consultation process measure. Fam Pract.

[13] Solaja O, Moloo H, Hopkins E (2022). Implementation, results and face validity of the Consultation and Relational Empathy measure in a Canadian department of surgery. Can J Surg.

[14] Nunnally JC (1978). An overview of psychological measurement. Clinical Diagnosis of Mental Disorders: A Handbook.

[15] (2022). Sample Size Calculator. https://clincalc.com/stats/samplesize.aspx.

[16] Howick J, Steinkopf L, Ulyte A, Roberts N, Meissner K (2017). How empathic is your healthcare practitioner? A systematic review and meta-analysis of patient surveys. BMC Med Educ.

[17] Al-Habbal K, Djoundourian A, Nassar E, Tayara Z, Mercer SW, Abi-Habib R (2022). Reliability and validity of the Arabic version of the Consultation and Relational Empathy (CARE) measure. Fam Pract.

[18] Heo R, Shin J, Kim BS (2023). Quantitative measurement of empathy and analysis of its correlation to clinical factors in korean patients with chronic diseases. Clin Hypertens.

[19] Maurici M, Arigliani M, Dugo V, Leo C, Pettinicchio V, Arigliani R, Franco E (2019). Empathy in vaccination counselling: a survey on the impact of a three-day residential course. Hum Vaccin Immunother.

[20] Keulen MH, Teunis T, Kortlever JT, Vagner GA, Ring D, Reichel LM (2020). Measurement of perceived physician empathy in orthopedic patients. J Patient Exp.

[21] Birhanu Z, Assefa T, Woldie M, Morankar S (2012). Predictors of perceived empathy among patients visiting primary health-care centers in central Ethiopia. Int J Qual Health Care.

[22] Walocha E, Tomaszewski K, Wilczek-Rużyczka E, Walocha J (2013). Empathy and burnout among physicians of different specialities. Folia Medica Cracoviensia.

[23] Pavlova A, Wang CX, Boggiss AL, O'Callaghan A, Consedine NS (2022). Predictors of physician compassion, empathy, and related constructs: a systematic review. J Gen Intern Med.

